# Supplementary material for: Are carbon emissions trading and green financial instruments synergistic? -Comprehensive quantitative research based on content analysis
Source: PLoS One. 2024 Mar 7;19(3):e0298601. doi: 10.1371/journal.pone.0298601 (PMC10919601; doi:10.1371/journal.pone.0298601)
Supplement: S1 Data — (ZIP) [file pone.0298601.s001.zip › Data/地级市Z_F工作报告.zip!/║┌┴·╜¡/┤≤╟∞╩╨/└┤╫╘╣1⁄2╓┌║┼í╛┬φ┐╦╩2╛▌═°í┐_2008.docx]

各位代表：

　　现在，我代表大庆市人民政府，向大会作工作报告，请予审议，并请市政协委员提出意见。

　　一、2007年工作回顾

　　过去的一年，我们按照市委确定的“抢抓历史机遇，力攀战略新高，实现大庆可持续发展新跨越”的总体思路，围绕发展经济、改善民生、促进和谐，积极推进48项重点工作和60件大事实事，圆满完成了市八届人大一次会议确定的任务。

　　（一）国民经济又好又快发展。全市实现地区生产总值1822.4亿元，增长10.5%。地方财政总收入117.6亿元，增长38.6%。完成固定资产投资481.6亿元，增长25.2%。工业经济速度、质量同步提升。完成工业增加值1506亿元，增长9.9%。生产原油4169.8万吨、天然气25.5亿立方米，石油工业、中直石化工业、地方工业分别实现增加值1185亿元、86亿元和185亿元，增长4.4%、29.5%和35%。石化项目建设取得突破，120万吨乙烯改扩建及配套工程、120万吨焦化装置等项目开工建设，20万吨醋酸等项目顺利投产，10万吨苯乙烯建成完工。园区承载能力不断增强，全市20个工业园区新开发面积18平方公里，大庆经济技术开发区、黑龙江软件园等园区建设进展顺利，新建续建地方工业项目202个（投资500万元以上），园区工业实现增加值113.2亿元，占地方工业61.2%。高新区宏伟园区成为国家火炬计划石化产业基地，林源园区成为新型复合材料及制品产业基地。节能减排取得实效，实施燃煤锅炉改造、区域热电联产等十大重点节能工程，爆破拆除龙凤热电厂13.3万千瓦发电机组，查处违法违规排污企业109家，依法关闭21家，17家国家和省挂牌企业、25家市县区挂牌企业全部完成整改任务，万元gdp综合能耗同比下降4.2%。日月星大豆工业园、肇源皮革工业园、红岗铁人生态园分别被推荐为国家和省级循环经济试点园区。农村经济稳步发展，大旱之年农民增收粮食稳产。农业增加值实现55.3亿元，增长3.9%。农业生产条件继续改善，新建抗旱保收田103.5万亩，新组建农机合作社87个。现代农业发展势头强劲，建设高标准现代化农田13.2万亩，培育绿色有机食品基地350万亩，绿色（有机、无公害）产品标识发展到218个；农民专业合作社发展到127家，实现销售收入6.7亿元。畜牧业稳步健康发展，深入实施“六吨奶”工程，健全畜禽改良和疫病防治体系，实现增加值28亿元。农业产业化进程加快，天泰马铃薯等9个农产品重点加工项目开工建设，省级以上龙头企业发展到16家，具备120万吨鲜奶、260万头生猪和162万吨粮油加工能力，农业产业化经营收入实现110亿元，带动基地户均增收4300元。第三产业提档升级。实现增加值218.8亿元，增长15.8%。商贸市场繁荣，沃尔玛、毅腾商都等大型商场建成使用，汽车、文化、娱乐等消费持续升温；“万村千乡”市场工程深入推进，建成配送中心9个，新增农家店360个，乡村覆盖率达85%。全市社会消费品零售总额达到302亿元，增长19.7%。现代物流产业加快发展，大庆石油物流园区初具规模。华拓数码等服务外包骨干企业蓬勃发展，大庆服务外包产业园被确定为中国服务外包示范区。高新区被批准为国家科技兴贸创新基地。旅游业实现总收入13.4亿元，增长13.5%。

　　（二）新农村建设扎实推进。坚持城乡统筹协调发展，加大农业基础设施和社会保障投入力度，积极实施“三通四建五保六帮”工程，农村面貌继续发生新的变化。农村基础设施建设步伐加快。深入推进通村屯交通工程，建设通村路480公里，通村屯硬化路面达到70%。继续实施农村饮水安全工程，建设饮水工程115处，铺设供水管网80万延长米，5.8万农民受益。大力推广清洁能源利用，7961户农村居民用上沼气，农村生活环境明显改善。农村社会事业持续进步。改造危房校舍46所、2.9万平方米，20万名农村学生免除学杂费，6万名学生免费领取教科书，1.2万名寄宿生得到生活补助。新建扩建8所乡镇卫生院，集中培训农村医疗骨干，建立市级医院和乡镇卫生院援助对接机制。加强农村文化基础设施建设，组建农民业余文化团体100个，建设农家书屋485个，成功举办“大地欢歌”农民艺术节，广播电视“村村通”新增农村用户5000户。农村社会保障体系不断完善。启动农村最低生活保障制度，4.5万人纳入低保范围，低保标准由683元提高到800元，发放低保救助金1623万元。巩固新型农村合作医疗制度，参合人数达到95.5万人，参合率94.8%，累计报销金额5200万元。集中救助五保户、优抚户、受灾户和贫困户，筹集下拨救灾救济资金822万元，8万户农民获得救助。

　　（三）现代都市建设迈出新的步伐。全力推进308个城建项目，建筑面积625万平方米，总投资97亿元。城市功能项目建设实现新跨越。飞机场、铁路大庆西站、东城区污水处理厂、油田科技博物馆、机场路等38项市级重点工程如期推进。大庆书苑、大庆歌剧院、青少年科技活动中心投入使用。奔腾立交桥、南三路、勤奋北路、纬十一路、让通线“平改立”等8路3桥竣工通车，“三纵三横”立体式交通格局初步形成。创建国家卫生城市取得新突破。集中组织开展整治市容市貌、五小行业、集贸市场、牌匾广告、食品卫生等五大战役，新建垃圾中转站2座、公厕110座，修建改造平房区道路96条、排水渠6万延长米；清除卫生死角5000多处，清运垃圾近百万吨，拆除违章建筑6000多处；对1.3万个五小行业企业实行脸谱公示分级量化管理，达标率由整治初的8.9%提高到93.5%；综合治理43座集贸市场，实行全天候驻场式管理，达标率由整治初的21%提高到90%以上；整治各类牌匾广告11万块，综合恢复率达到90%以上；全市40个平房区、9个城中村、105条街道实现了卫生保洁全覆盖。创卫工作顺利通过国家暗访和技术评估。城市生态景观建设取得新进展。加强水环境综合治理，改造乘风湖等湖泡4个。突出搞好城区绿化美化，新建油田广场、果午生态广场等生态园7个，启动百里绿色油田示范带，推进道路景观绿化建设，新建绿地692.8万平方米，植树造林186.7万株，建成区绿化覆盖率达37%，人均公共绿地面积9.5平方米，城市人居环境不断改善。城市经营管理取得新成效。完成土地收储16宗、484公顷，土地收益24.2亿元，位居全省之首。在全省唯一获得出租车经营权有偿出让批复，公开拍卖2000台出租车经营权，实现收益1.2亿元。集中开展铁路沿线综合治理，动迁沿线居民1.2万户，拆除各类建筑108.7万平方米，植树绿化400公顷，铁路沿线环境明显改观。加快建设数字城管平台，实现市区网络互联、信息共享，城市精细化、现代化管理水平不断提高。

　　（四）城乡人民生活水平继续提高。城镇居民人均可支配收入、农民人均纯收入分别达到15570元和3917.7元，分别增长7.1%和8.5%。统筹城乡就业。全年累计安置就业4万人，开发公益性岗位1380个，城镇登记失业率控制在4%以内。优先安置下岗失业和困难家庭人员，实现零就业家庭“动态归零”。积极培育大庆劳务品牌，稳定转移农村劳动力22万人，实现劳务收入16亿元。完善小额贷款担保政策，累计为957名下岗失业人员和农民发放贷款2790万元。健全保障体系。积极开展“五险”扩面，净增32.8万人次，收缴保险金18.9亿元，在全省率先实现覆盖城乡的全民医保。低保救助内容扩充到“十位一体”，6.5万人得到救助，发放低保救助金1.1亿元。改善居住条件。完成龙凤澳龙、让胡路铁路平房区等12处棚户区拆迁改造，落实补偿资金1.8亿元。建设惠民苑一期等经济适用住房11.2万平方米，1760户困难群体有了住房保障。完善廉租房保障制度，市区699户居民受益。累计归集住房公积金136.9亿元，发放贷款39亿元，均居全省首位。完成10万户天然气入户工程，置换通气5万户。安排价格调节基金1857万元用于居民液化气补贴。规范市场秩序。严厉打击“七种”违法车辆，综合治理超速超载等违法违规行为，累计查扣各种车辆5546台次、依法销毁1926台。新增诚信平价药店11家，推行农村药品“一价制”，减轻群众医疗负担2500万元。加强食品安全专项整治，城市食品安全消费满意度综合评价全省第一。药品市场、建筑市场和农资市场等整治行动深入推进，市场管理秩序明显好转。建设平安大庆。强化治安防控体系建设，严厉打击各种违法犯罪活动，8类主要刑事案件同比下降28.5%。落实安全生产责任制，事故数量、伤人、亡人和经济损失四项指标整体下降。坚持领导干部信访接待日制度，依法、妥善解决群众合理诉求，人民群众满意度进一步提高。

　　（五）社会事业不断繁荣进步。教育事业优先发展。靓湖学校、万宝三区小学教学楼建成使用，奥林、三永等新建学校完成前期准备，维修改造市区50所薄弱学校，“两基”工作通过国家验收。高等教育在校生规模扩大到6.6万人，优质高中招生比例达到74.6%，高考成绩保持全省领先。优化整合职业教育资源，合并迁建萨区职高和建设中专，全市中等职业学校在校生达到1.5万人。医疗卫生水平整体提升。加强综合医院建设，培育发展特色专科，油田总医院住院三部、市中医院移址新建工程开工，6项医学科研成果达到国内先进水平。继续推行三级医院领办社区模式，建立社区卫生服务机构188个，覆盖面达到96%。加大疾病预防控制力度，重点在食品卫生、饮水安全等方面加强监督管理，提高公共医疗卫生服务水平。文化体育全面繁荣。“书香大庆”、“大庆之冬”、“激情之夏”、“礼仪大庆人”、“全民健身与奥运同行”等活动广泛开展。话剧《铁人轶事》获“五个一工程”入选作品奖，《天鹅湖》、《蓝色多瑙河》等高雅剧目走进大庆，歌舞秀《经典永恒》广受赞誉，我市运动员在国家级以上体育赛事中获得奖牌60枚。与中央电视台成功举办“奥运城市行•走进大庆”节目，展示了大庆市民喜迎奥运的精神风貌。精神文明建设深入开展，双拥工作富有成效，我市第5次被命名为全国双拥模范城称号。科技创新取得突破。开发建设科技项目384个，转化科技成果153项，69个项目列入国家和省科技计划，科技创新对经济增长贡献率达到47%。企业自主创新取得突破，节能降耗成效明显，油田公司荣获国家工业领域最高奖项“中国工业大奖”，总经理王玉普当选中国工程院院士。

　　（六）改革开放继续深化。重点领域改革稳妥推进。规范政府采购程序，建立网上申报、项目催办和跟踪监督制度体系，完成采购项目3477个，节约财政资金1.7亿元。深化教育改革，农村义务教育经费保障机制初步建立，全市高中课改全面启动，高中教育质量评价制度得到健全完善。国有企业改革步伐加快，粮食企业、自来水公司、大庆宾馆、客运集团等单位体制机制改革继续深入，组建了交通投资公司、让胡路火车站地区投资开发公司等，现代企业管理制度进一步完善。大庆市商业银行吸纳中粮集团入股，拓展农业金融和能源金融业务，企业竞争实力显著增强。全市公务员登记工作全面完成，登记合格率达到93.4%；市区机关事业单位工资顺利改行，人均月增资342元。对外开放取得较大突破。全市招商引资实际到位资金110.8亿元，增长4.7%，连续3年突破百亿元大关，引进港荣铝业等亿元以上项目42个。实现进出口贸易总额6.8亿美元，增长53.2%；全年实际利用外资9267万美元，增长109.2%。企业融资能力显著增强。加强上市企业培训指导和政策支持，三维科技、大丰油科等3家企业在境外成功上市，全市已有上市企业9家，累计融资34亿元。

　　（七）政府自身建设不断加强。政务公开取得突破。行政服务中心投入使用，46个部门、473个行政事项和23个服务项目进驻中心，削减审批环节2088个，累计办理事项34万件，办结率达到97%。扩大政务公开范围，全面启动教育、医疗、供热等公用事业单位办事公开，政务公开透明度和公共服务能力明显增强。效能监察普遍开展。举办首届政府工作论坛，开辟《市民议事厅》电视栏目，实施行风评议现场直播，形成政民互动良好氛围。切实发挥市长热线作用，受理群众来访来信来电11.3万件次，立案1.2万件、办结率100%，利益诉求表达渠道更加顺畅。建立勤政廉政测评体系，完善目标考核评价制度，实行行政绩效考核，政府机关行政效率逐步提高。民主法治深入推进。自觉接受人大法律监督和政协民主监督，认真办理人大代表建议275件、政协委员提案157件。广泛吸纳社会各界意见，征集人民建议10个方面723件，政府决策更加科学民主。行政执法行为渐趋规范，依法行政水平进一步提高。依法完成社区居委会换届选举，基层自治逐步完善。

　　此外，工商税务、邮政通讯、新闻出版、民族宗教、文史档案、外事侨务、审计统计、海关商检、国防人防、计划生育、老龄工作等也取得较大成绩。

　　各位代表，过去一年成就的取得，主要得益于市委的正确领导、科学决策；得益于人大、政协和社会各界的监督支持、鼎力相助；得益于石油石化企业、驻庆中省直单位同心同德、并肩创业；得益于全市人民团结一心、艰苦奋斗。在此，我代表市政府，向所有为大庆经济发展和社会进步做出贡献的同志们、朋友们，表示崇高的敬意和衷心的感谢！

　　各位代表，回顾过去一年的工作，我们深刻体会到，推进大庆可持续发展，必须坚持以科学发展观统揽经济社会发展全局，发扬大庆精神、铁人精神，推进经济社会又好又快发展；必须坚持民生为本民利至上的施政理念，切实实现好、维护好、发展好人民群众的根本利益；必须坚持用现代都市的理念规划建设城市，打造适宜人居和创新创业的城市环境；必须坚持深化改革扩大开放，构建适应市场经济需要的动力机制和体制保障；必须坚持深入推进地企协调发展，始终不渝地贯彻责任共担、发展共促、稳定共抓、城乡共建的战略方针；必须坚持解放思想，与时俱进，加快转变政府职能，更加注重市场监管、社会管理和公共服务，全力加快和谐社会建设。

　　各位代表，回顾过去一年的政府工作，我们也清醒地认识到，政府工作及自身建设仍有差距。主要是，支撑可持续发展的非油企业成长不够，名牌产品较少，油气、石化和农产品加工等产业链条不长，节能减排任务相对繁重；经济发展环境有待优化，创新创业氛围不够浓厚；县域经济发展基础比较薄弱，农民增收渠道单一、增长幅度不大，新农村建设任务还很艰巨；社会公共产品供需不够平衡，就业、教育、医疗、住房等民生问题仍很突出；政府一些部门和工作人员服务意识不强，执法水平不高，抓落实力度不够，领导推进科学发展的意识和水平有待进一步提高，等等。对于上述这些问题，我们将高度重视、深入研究，在今后工作中着力加以解决。

　　二、2008年工作安排

　　2008年，是全面贯彻落实党的十七大战略部署的起步之年，是我市实施“十一五”规划的承上启下之年，也是推进大庆可持续发展新跨越的关键之年，有机遇更有压力和挑战，任务极其繁重。我们必须坚持科学发展方向不动摇，坚持解放思想、与时俱进、深化改革不动摇，坚持改善民生理念不动摇，坚持建设现代都市目标不动摇，坚持城乡统筹发展、地企和谐共建的工作思路不动摇，通过坚持不懈的努力和扎实有效的工作，为实现可持续发展新跨越奠定坚实基础。

　　2008年政府工作总的要求是：全面贯彻党的十七大精神，深入落实科学发展观，按照市委七届三次全会确定的战略部署，以发展经济、改善民生、促进和谐为主题，全力支持百年油田建设，大力发展石化、农产品加工、装备制造等为重点的接续产业，努力改善发展环境和人民生活质量，推动城市与农村、经济与社会、产业与生态、地方与大企业协调发展，确保大庆可持续发展新跨越取得阶段性成果。主要预期经济指标是，地区生产总值增长8%，固定资产投资增长20%，城镇居民人均可支配收入和农民人均纯收入分别增长6%、11%，城镇登记失业率控制在4.5%以内，居民消费价格总水平涨幅控制在4.5%以内，万元gdp综合能耗下降4%。

　　（一）转变经济发展方式，加快新型工业化进程。探索实践产业基地化、企业集群化、产品名牌化、方式集约化、生产信息化的工业发展模式，提升工业经济整体水平，全市地方工业增速35%以上。加快建设优势产业基地。支持石油企业全力创建百年油田，扩大油气勘探范围，增加可采储量，原油开采4010万吨、天然气31亿立方米，夯实提升国家石油产业基地。利用中石油产业政策，推进大项目，搞好深加工，支持石化企业搞好120万吨乙烯改扩建及配套工程、30万吨聚丙烯新建等重大项目，加快建设国家石化产业基地。充分发挥农业资源富集优势，整合放大乳肉皮毛产业为主的畜产品深加工集群、米豆产业为主的粮食深加工集群、苇麻产业为主的纸纺深加工集群，抓好展华120万吨玉米深加工等大项目，全力打造国家重要农产品加工基地。依托油化企业，突出钻探、采油和炼化三大领域，抓好管理局抽油机、减速器和真空加热炉等已开工项目，规划建设潜油电泵、螺杆泵等一批产能扩建项目，举办石油石化装备制造业发展论坛，组建装备制造业企业联合体和工程中心，启动建设国家石油石化装备制造业基地。加强园区基础设施建设，调整优化园区产业布局。发挥高新区、经济技术开发区龙头作用，高标准、高起点，引大项目、建大企业；整合石油石化大企业所属中小企业、区属园区的生产要素，联大靠强，扶优扶强，大力发展石化和装备制造业。四县工业园区围绕农产品加工，挖掘优势、壮大规模、延长链条、提升层次。全力抓好地方工业项目建设。推进地方工业“百项工程”，重点搞好港荣20万吨铝材、瑞好5万千瓦风力发电和三精30亿只药用玻璃制品等项目建设，力争新建续建项目150个以上，销售收入10亿元以上的企业达到8家。切实抓好节能减排。改造东城区、八百垧和乙烯等污水处理厂，新建陈家大院泡和大同区污水处理厂，启动四县生活污水处理厂和生活垃圾无害化处理场，提高污水污物处理能力。搞好循环经济试点，推广利用太阳能、汽车燃气等新能源。切实增强科技创新能力。围绕主导产业，打造创新平台，支持石油石化企业开展技术创新，提升现有科研院所研发实力，加快建设石油石化装备制造研究院、石油石化产品深加工研究院和黑龙江省中加食品研发中心。依托创新载体，引进高级管理人员创办领办高新技术企业，支持高新区申报建设国家创新型科技园区。

　　（二）统筹城乡发展，扎实推进新农村建设。深入推进“三通四建五保六帮”工程，实施促农增收、设施建设、道路畅通、饮水安全、改善生态、造林绿化、医疗惠民、教育振兴、低保救助和市场流通“十项行动”，扎实做好“三农”工作，促进农村经济社会和谐发展。发展现代农业。加强基础设施建设，建设抗旱保收田103万亩、高标准膜下滴灌农田23万亩，新建农机合作社77个。调整农业结构，增加特色作物、经济作物种植面积，壮大优势产业带，发展优势产业村，粮经饲比例达到60︰23︰17。稳步提升畜牧业，抓好草原禁牧、品种改良、疫病防控和风险防范等项重点工作，加快发展质量效益型、环保型、生态型和集约型畜牧业，畜牧业增加值增长10%以上。推进农业产业化，扶强做大金锣、日月星等龙头企业，延伸产业链条，鼓励标准化生产，扩大绿色有机食品市场销售份额，培育跨区域农产品专业批发市场，市级以上龙头企业达到54家、带动基地25万户。建设现代农村。加快发展农村社会事业，建设四级农村公路500公里，维修改造危旧校舍35所，完善110个辅助教室，新增标准化农家连锁店200家，解决5.8万居民饮水难、2.4万户低保家庭就医难和就学难问题。实施“一建三改”，建沼气池、改厨、改厕、改圈，新增沼气入户1万户，加快转变农村生活方式。推进卫生信息、卫生支农和乡镇卫生院标准化建设工程，改善农村医疗卫生条件。搞好村镇规划建设，突出抓好中心村和小城镇，优先发展乡镇所在村、交通要塞村和流通活跃村。落实好强农惠农政策，化解乡村不良债务和农村义务教育历史债务，增强农村后续发展能力。培育现代农民。强化农民技能培训，推进科技培训工程，培养有文化、懂技术、会经营的新型农民，增强生存、就业和发展的综合素质。健全科技推广体系，完善专业技术人员、大中专毕业生到农村工作政策。加快发展各类农村合作经济组织，提高农民抵御市场风险能力。

　　（三）抓住关键突破重点，加快服务业提档升级。打造高档商贸业。力争引进“大福源”、“家乐福”入驻我市，打造品牌商企，建设精品商区，构筑特色商圈，促进商贸业提档升级。改造建设东安市场、龙宫、红岗商贸中心和让区北方市场等重要商贸街区，规划建设集商贸、餐饮、休闲、娱乐等于一体的大型卖场。推进“超市进社区”，拓展连锁经营领域，促进社区便捷消费、安全消费。积极发展物流业。完善大庆石油物资物流园、港桥物流园基础功能，培育发展万宝商都物流园，规划开发空港物流园，建设粮食物流园，扶持小商品批发、旧物交易等市场拓展辐射空间，提升经营层次。大力发展服务外包业。用好用活示范区各项政策，完善高新区服务外包园、黑龙江软件园等园区功能，增强产业承载力和市场辐射力。紧盯国内外重点服务市场，加大招商引资和产业承接力度，推动产业关联发展。做大做强华拓数码、华创电子和三维集团等骨干企业，大力发展石油石化工程技术服务、软件开发等优势产业，壮大服务外包企业实力。创新发展金融业。优化金融生态环境，增强吸储放贷能力，促进金融与经济有机融合。积极引进外资银行、保险和信托等机构，促进金融多元发展。支持商业银行上市融资，逐步实现股本多元化。引导鼓励金融机构向农村发展，丰富金融业务和产品，加大农村经济支持力度。加快发展旅游业。坚持政府引导、社会参与、市场运作、保护开发、突出特色，办好中国（大庆）首届湿地文化节。规划建设景点、场馆等基础设施，精心打造石油文化、生态湿地、民族风情等特色旅游产品，宣传推介铁人故里、百湖览胜、湿地人家等主题景区，全力做好省内外旅游促销活动，确保把湿地文化节办成国内具有一定影响力的品牌节庆盛会。

　　（四）创新理念提升层次，进一步推进现代都市建设。按照现代自然理念规划城市。贯彻落实《城乡规划法》，接轨国内国际领先建设标准，突出“现代、自然”风格，完善总体规划、详细规划，做好城市设计，确保规划的连续性、指导性和权威性。加快建设都市枢纽工程。东部城区整体推进萨尔图飞机场工程建设，力争明年机场试航。西部城区启动建设铁路大庆西站，搬迁“后四厂”，建设新城区。中部城区按照国家一级公路客运站标准，扩建中七路长途客运站。开工建设治理两大水系。东部城区黎明河流域水系，打通萨北湖、滨州湖和万宝湖等5个湖泊，启动建设东城水库，彻底改造黎明河，使北起红旗渠、中通各湖泊、南至龙永路的河岸环境得到根本改善。西部城区从大庆水库和龙虎泡水库引水，经西排干和中央排干将月亮泡、果午泡、碧绿湖、明湖、让胡路泡等32个湖泡连通，构筑起西部水系网络，逐步建成休闲游览胜地。大规模实施城市绿化工程。打造绿色景观区，对红旗地区、青龙山地区、五湖生态园、中兴北园等区域进行森林式绿化；建设景观大道，对世纪大道、铁人大道、中三路、南三路、经九街、机场路等主干路整体绿化；推进油田绿色长廊建设，支持大企业对北起采油六厂、南到采油五厂的产能区，进行生态环境改造，力争用5年时间建成靓丽优美生态长廊。抓好城建重点工程。规划论证哈大齐城际客运专线、城市轨道交通、大广高速大庆段和萨大路改造等项目，新建续建让通线平改立、五星级宾馆、万人体育馆、龙宫地区和街心花园改造等工程，按照国家“5a”级旅游标准，启动规划建设北国之春温泉城。继续加大创卫推进力度。完善城区基础设施，加快建设生活垃圾无害化处理场和生活污水处理厂，启动城市绿色再生资源回收工程，新增垃圾中转站和公共厕所各10座，完善城市污水污物排放与收集系统。加快城中村改造步伐，启动推进让胡路富强铁东、高新区东风农场青龙屯等城中村和萨尔图铁东、龙凤南砖厂等棚户区改造。建立创卫长效管理机制，继续开展市容市貌、五小行业、集贸市场等集中整治，严格落实门前“四包”，开展卫生区、卫生镇、卫生村和卫生街道等评比活动，健全监管、考评、激励等机制办法；推进数字城管工程，扩大城管监控探头覆盖面，全面提高城市网格化、数字化、精细化管理水平。确保年内在全省率先获得国家卫生城市命名。

　　（五）优化发展环境，拓展改革开放广度和深度。构筑体制机制新优势，激发释放经济发展活力。深化各项改革。按照国家的统一部署，整合行政机构。推进事业单位改革，完善改革总体方案和配套政策，搞好教育文化、机关后勤和驻外办事处等单位改革工作。继续深化国企改革，推进投资、担保、资产经营类行业的国有资本优化重组，深化企业用工、分配制度改革，逐步推行企业经营者任期制和年薪制。深化国有资产监管、财政投资评审、财政集中支付、政府采购和县乡财政管理体制改革，打造新的运行机制。推进招商引资。准确把握产业、市场和要素走势，实施理性选资招商、园区专业招商和产业链式招商，重点引进成熟稳定、产业链长、生态环保型大项目，加快招商方式转型。开展企业经理人论坛、大庆招商峰会等大型活动，提高招商层次质量。调整招商相关部门职能，发挥产业策划中心作用，提高招商能力水平。全年招商引资额保持100亿元以上。抓好融资上市。制定企业上市培育计划，优化服务平台，完善扶持政策，逐步建立良性互动、梯次发展的培植体系。争取肇融亚纺、鑫龙化工和瑞好能源等企业成功上市，打造“大庆板块”。努力把大庆高新区纳入国家首批非上市股份公司股权转让代办系统试点。优化发展环境。实施中小企业创业成长工程，研究制定发展奖励政策，对经济规模、市场占有率、税收贡献、科技进步、品牌培育方面贡献突出的地方企业，给予物质奖励，鼓励非公有制经济扩张发展。调整人才政策，探索实施以技术、知识产权等生产要素参与的投资制度，人才贡献与薪酬、生活待遇和社会地位挂钩的奖励制度，不断优化人才成长环境，壮大企业人才、科技人才、技能人才、社会人才和机关人才队伍。发展对外贸易。加快出口加工区、内陆港、保税仓库建设，优化外贸出口环境。抓住我省对韩经贸科技合作战略升级机遇，开展交流合作，扩大对韩贸易。壮大外贸骨干企业，培育出口产业，推动国际化经营，增强企业国际业务拓展能力，力争外贸进出口总额突破8亿美元，增长20%以上。

　　（六）着眼建设和谐社会，努力保障和改善民生。抓好覆盖全市的城乡就业。贯彻落实《就业促进法》、《劳动合同法》，全面推进统筹城乡就业试点工作。加强就业和创业培训，将创业培训范围扩展到高校毕业生、退役军人和农村富余劳动力等社会群体。开展“充分就业社区”创建活动，积极落实社会保险补贴、小额贷款等就业政策，城镇新增就业3.4万人、稳定转移农村劳动力24万人。完善惠及城乡的社会保障体系。实施社会保险惠民工程，继续扩大“五险”覆盖面，让更多劳动者进入“安全网”。积极争取国家政策支持，推进城乡全民医保，进一步扩大医保覆盖面。启动农村居民养老保险试点，探索实施基金多元结构、标准基本适度、兼顾各方利益、保障平稳过渡的新型农保办法，逐步实现农民老有所保。完善城市“十位一体”综合救助体系和农村低保综合救助体系，提高弱势群体生活质量。建设市民满意的和谐社区。理顺社区组织关系，明晰社区居委会职责，探索推行居站分设、选聘分开。加大市区两级财政投入力度，完善社区基础设施，建设社区活动场所55个。确保业主委员会等自治组织发挥自治功能，让居民政治上得到权利、物质上得到实惠、精神上得到快乐。改善群众生产生活环境。严格控制商品房开发地域和规模，高标准完善小区功能，房产开发控制在300万平方米以内。抓好惠民苑二期、让胡路铁路等经济适用住房规划建设，建筑面积30万平方米。发挥住房公积金作用，降低贷款门槛，提高缴交比例，扩大覆盖范围。继续抓好铁路沿线综合整治，争取3至5年城中村改造全部完成。完善小区停车场、公共停车场建设，疏导维护交通秩序。落实城乡免费义务教育政策，加强校园周边治理，规范校园午餐管理，创造良好环境。强化市场物价监管，保障重要消费品市场供应，建立低保家庭生活补贴与物价上涨联动机制。加强小区物业监管，搞好食品药品、交通运输、建筑建材、文化和保险等市场整顿，维护群众利益。

　　（七）创建全国文明城市，全面繁荣各项社会事业。着力提升政府公共服务水平，提高市民文明健康素质，加快和谐社会建设步伐。突出解决教育公平问题，均衡发展教育事业。巩固提高“两基”成果，市区全部通过“双高普九”验收。搞好世纪阳光等新建小区学校配套建设，完成40所薄弱学校改造任务。推进素质教育，减轻学生课业负担，健全完善中小学综合办学水平评估体系。开展德育工作年创建活动，选树“十百千”德育典型。大力发展职业教育，规划建设市区公共实习实训基地，整合技工教育资源，打造焊接、汽修等国家级名牌专业，争取杜蒙职教中心列为国家重点支持项目、肇源职教中心等学校列为省级重点扶持项目。积极发展特殊教育，建设集康复、教育、培训于一体的残疾人发展中心。结合城市发展实际，深化教育资源整合，优化学校布局。加强公共医疗卫生建设，提高居民健康水平。推进重点专科建设，支持8所市级医院打造医疗特色服务中心，逐步达到国内同类先进城市水平。提高社区卫生服务质量，保持新农合参合率，提高统筹额和补偿标准。健全公共卫生服务体系，提高疾病防控能力，保障群众健康安全。繁荣发展文体事业，丰富群众文化生活。规划论证世界石油公园、文化创意园，扩展都市文化内涵。发挥歌剧院、青少年科技活动中心和博物馆等载体功能，加强文化产品、演出等市场开发，繁荣壮大文化产业。开展百优剧（节）目下乡活动，办好第三届“大地欢歌”农民艺术节，丰富群众文体生活。创排电视剧《脊梁》和大型生态舞剧《鹤鸣湖》，举办好中国大庆铁人极限挑战赛，高质量完成奥运火炬传递系列活动。提高社会文明程度，创建全国文明城市。以弘扬大庆精神、铁人精神为主线，全面加强社会公德、职业道德、家庭美德和个人品德建设。深入开展全民读书活动，做好科普工作，推进文明社区、文明行业、文明单位和文明窗口创建活动。加强文明村镇建设，树立农村和谐文明新形象。对照全国文明城市“六大环境、一个创建活动”测评体系，突出解决城市环境、市民素质等方面存在的问题，力争大庆成为全国文明城市。

　　（八）大力支持石油石化企业发展，促进地企深度融合。继续清理占压油气管线建筑，加大油田治安综合治理力度，加强产能区生态治理恢复，营造良好生产环境。支持石油管理局在油城区内开展地震采集工程，协调解决勘探施工中遇到的实际问题。协助做好外围油田职工回迁，搞好政策跟进、土地规划、审批服务和配套建设。强化市场监管体系建设，推进产业有序衔接、要素有序流动、竞争有序进行。支持企业壮大主营业务，实现持续健康发展。地企协作领域要有新突破。共同研究国家发展接续产业政策，争取资源型城市可持续发展试点政策，落实省政府与中石油战略框架协议政策。争取启动盘活林源炼油厂，联手搞好大乙烯吃配、石油装备制造等大项目建设，逐步扩大石化原料、人才技术、物资采购和资产盘活等方面的融合度，促进生产要素共享共用。同步推进企业节能减排和清洁生产，搞好小油田开发和天然气综合利用，发展壮大地方接续产业。共同规划建设城市基础设施、企业生产生活配套设施，整治城市生活环境，搞好大企业职工生活基地建设。协作开发就业岗位，加强社区建设管理，搞好矛盾排查调处，全力维护社会稳定。地企协调机制要有新拓展。完善地企高层联席会议制度，共同研究制定政策、推进重点工作、破解发展难题。加强基层衔接，缩短流程环节、提高办事效率，逐步形成上有高层协商、下有基层落实的新型地企协作共建机制。

　　（九）完善民主法制建设，努力实现社会和谐稳定。深入推进依法治市，开展“五五”普法宣传教育，维护公平正义，提高社会民主化、法治化、文明化程度。加强民主政治建设。自觉接受人大监督，主动加强与人民政协的联系，及时办理人大代表建议、政协委员提案，认真听取各民主党派、工商联和无党派人士的意见，支持工会、妇联、共青团等群团组织开展工作。完善社区管理和服务体制，加强农村基层民主建设，推进依法自治，保障居民知情权、参与权、表达权和监督权。维护群众合法权益。发挥市长“12345”、社区“96760”、法律“148”等维权服务热线作用，畅通民意诉求渠道。加大政务、厂务和村务公开力度，保证公民民主权利。开展人民建议征集活动，广泛听取社情民意。切实维护老人、妇女、儿童、残疾人和失地农民等弱势群体权益。提高社会管理水平。健全基层社会管理体制，强化社会组织功能，形成党委领导、政府负责、社会协同、公众参与的管理格局。制定出台利益协调、诉求表达、矛盾调处、权益保障“四个机制”实施办法，确保社会管理更加规范化、制度化和实效化，科学协调和分配公共利益。加强社会诚信体系建设，规范社会主体行为，营造诚实守信、文明有序社会环境。搞好平安大庆建设。坚持“打防结合、预防为主”，加强社会治安综合治理，推进“三基两化”建设，新建1000个监控探头，全面提高人防、物防、技防和联防水平，初步建成城市安全监控网络。完善道路街区标识，严打七种违法车辆，巩固“平安畅通县区”创建成果，打造大庆“平安”品牌。全力做好信访工作，及时化解不稳定因素。完善突发公共事件应急预案，提高应急处置能力。坚持安全发展理念，强化基础设施，健全安全机制，遏制重特大事故发生，保障群众生命财产安全。

　　（十）提高政府行政效能，增强公共服务能力和水平。深入开展“行政绩效年”活动，切实加强政府自身建设，加快转变政府职能。提升行政能力，建设服务政府。推进政府服务重心下移，合理划分市、县（区）事权。创新完善行政服务中心、政府工作论坛和市民议事厅等载体功能，畅通政民“绿色通道”。启动城市信息化建设工程，推进电子政务和电子监察。进一步精简审批事项，压缩审批流程，提高办事效率和服务质量。规范运行程序，建设法治政府。完善行政决策机制，落实政府工作规则，坚持依法、民主、科学决策。理顺行政许可执法体制，进一步明确市、县（区）、乡镇和街道行政许可权限。大力推进政务公开，提高政府重大决策、重点领域和重要事项透明度。推进城管、文化和农业等领域综合执法，整合执法资源，统一执法力量，规范行政处罚自由裁量权，提高整体执法效能。解决热点难点问题，建设责任政府。高度重视改善民生，把工作重点、政策支持重点和财力保障重点放在社会管理和公共服务上，切实为人民负责。关注热点、破解难点，全力解决好市场监管、环境整治、房屋拆迁、就医就学和就业安置等方面突出问题。严格落实行政问责、首问负责和过错追究等项制度，加强政府部门检查考核，有责必问、有岗必查、有错必究。求实务实落实，建设效能政府。加强决策执行落实，做到有令坚决服从、无令主动支持。强化绩效考核，推行iso9000质量管理认证体系，科学评价政府工作绩效。搞好跟踪督办，保证政府大事实事和重点工作有效推进。全面加强机关作风建设，开展解放思想、与时俱进大讨论活动，引导推动机关工作人员以开拓进取的精神和创新创业的激情推进工作，形成想事干事成事氛围，崇尚敬业卓越，反对虚功庸为。开展创建政府系统发展型领导班子活动，不断提高推进科学发展的能力。健全制约机制，建设廉洁政府。强化行政效能监察，整合产权交易、土地出让、建设工程招投标和政府采购，搭建统一的公共资源交易平台。认真执行廉政建设各项规定，继续开展治理商业贿赂，严格公务人员管理，增强拒腐防变能力。严肃财经纪律，推进阳光审计、效益审计，避免违法违规、铺张浪费。

　　各位代表，推动大庆可持续发展的使命崇高而神圣，我们肩负的责任重大而光荣。我们要在市委的正确领导下，以党的十七大精神为指针，深入落实科学发展观，弘扬大庆精神和铁人精神，团结依靠全市各族人民，解放思想、与时俱进，抢抓机遇、开拓进取，为实现可持续发展新跨越宏伟目标而努力奋斗！
